# Materials characterisation by angle-resolved scanning transmission electron microscopy

**DOI:** 10.1038/srep37146

**Published:** 2016-11-16

**Authors:** Knut Müller-Caspary, Oliver Oppermann, Tim Grieb, Florian F. Krause, Andreas Rosenauer, Marco Schowalter, Thorsten Mehrtens, Andreas Beyer, Kerstin Volz, Pavel Potapov

**Affiliations:** 1 Institut für Festkörperphysik, Universität Bremen, Otto-Hahn-Allee 1, 28359, Bremen, Germany; 2 Faculty of Physics and Materials Science Center, Philipps Universität Marburg, Hans-Meerwein-Straße, 35032, Marburg, Germany; 3 GLOBALFOUNDRIES Dresden Module 1, Wilschdorfer Landstraße 101, 01109, Dresden, Germany

## Abstract

Solid-state properties such as strain or chemical composition often leave characteristic fingerprints in the angular dependence of electron scattering. Scanning transmission electron microscopy (STEM) is dedicated to probe scattered intensity with atomic resolution, but it drastically lacks angular resolution. Here we report both a setup to exploit the explicit angular dependence of scattered intensity and applications of angle-resolved STEM to semiconductor nanostructures. Our method is applied to measure nitrogen content and specimen thickness in a GaN_x_As_1−x_ layer independently at atomic resolution by evaluating two dedicated angular intervals. We demonstrate contrast formation due to strain and composition in a Si- based metal-oxide semiconductor field effect transistor (MOSFET) with Ge_x_Si_1−x_ stressors as a function of the angles used for imaging. To shed light on the validity of current theoretical approaches this data is compared with theory, namely the Rutherford approach and contemporary multislice simulations. Inconsistency is found for the Rutherford model in the whole angular range of 16–255 mrad. Contrary, the multislice simulations are applicable for angles larger than 35 mrad whereas a significant mismatch is observed at lower angles. This limitation of established simulations is discussed particularly on the basis of inelastic scattering.

Scattering experiments are fundamental in modern physics to investigate structure, strain, bonding, disorder, temperature and chemical composition of solids. Owing to their characteristic scattering cross sections these properties can leave distinct signatures within the angular distribution of scattered radiation. Due to its excellent spatial resolution of up to 50 pm, scanning transmission electron microscopy (STEM) emerged as a prominent technique to probe, e.g., chemical composition^1^,^2^, strain^3^,^4^ or structures containing light atoms^5^,^6^,^7^. However, by commonly using a ring-shaped detector both annular dark and annular bright field STEM are widely insensitive to the angular distribution of scattered intensity because it is integrated over a full ring azimuthally and a broad intervall radially. This typically covers a few hundred milliradians in Z-contrast STEM. Though this setup enables data collection at 10^6^–10^7^ positions of the electron probe at dwell times in the microsecond range, it drastically limits the versatility of STEM as the characteristic angular dependence of scattered intensity collapses to a single value.

In this work we report on angle-resolved STEM (ARSTEM) imaging, which is able to probe the angular dependence of scattered intensity under established STEM conditions as to solid-state characterisation and scattering theory verification. In particular, we first present the independent measurement of composition, thickness and strain in a GaN_x_As_1−x_/GaAs layer by exploiting two dedicated angle intervals of 42–66 and 82–141 mrad. In this way, we overcome a fundamental limitation of composition quantification by high-angle annular dark field (HAADF) STEM which involves the interpolation of the specimen thickness from regions with known composition. Secondly, we measure the strain-, thickness and composition-dominated contrast as a function of the scattering angle in a Ge_x_Si_1−x_/Si metal-oxide semiconductor field effect transistor (MOSFET) and discuss its compatibility with theoretical results, namely Rutherford scattering and the multislice approach.

By positioning a software-controlled, motorised iris-type aperture with variable radius directly above a conventional annular detector, we developed a simple setup that still exploits the excellent speed and quantum efficiency of present hardware. In particular, an image corresponding to a dedicated angle interval *β*  ∈ [*β*_1_, *β*_2_] is obtained from


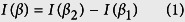


with images I(*β*_1,2_) taken at iris radii of *β*_1_ and *β*_2_, respectively. Although ultrafast cameras^8^,^9^,^10^,^11^,^12^,^13^,^14^,^15^ are currently introduced to the field of low-angle STEM, our approach exhibits several advantages. As quantitative analyses of atomically resolved STEM intensities rely on Voronoi diagrams^16^, Gaussian mixture models^17^,^18^ or probe integrated cross sections^19^, each atomic column must be sampled with a sufficient number of probe positions. Here, acquisition of the 2 K × 2 K data used for the measurement of composition, thickness and strain in GaNAs at atomic resolution took 5 min 40 s. Even with a pixelated, ultrafast detector operating at 2 kHz this recording would have taken 35 min and more than one hour at the more typical frame rate of 1 kHz. Moreover, we optimised the recordings such that intense low angle data are recorded faster than weak high angle data whereas direct detectors partially suffer from their limited dynamic range^8^,^9^,^14^ or radiation hardness. Furthermore one is often interested in the signal integrated over a few dedicated angular intervals, so that the iris approach efficiently reduces the data to a manageable amount and overcomes the limited angular resolution and flexibility of multi-ring detectors^20^,^21^ or strips of multiple apertures with fixed geometries^22^. It has similar advantages over the combination of several circular or annular detectors with different radii possibly available simultaneously in one microscope. Note that changing the camera length of the microscope is usually possible in fixed steps only and does not allow imaging at dedicated detector acceptance angles. Finally basing ARSTEM on annular detector design maintains the possibility to capture low-angle data with a spectrometer simultaneously.

## Results

### Composition, thickness and strain in GaNAs

**Figure 1:**
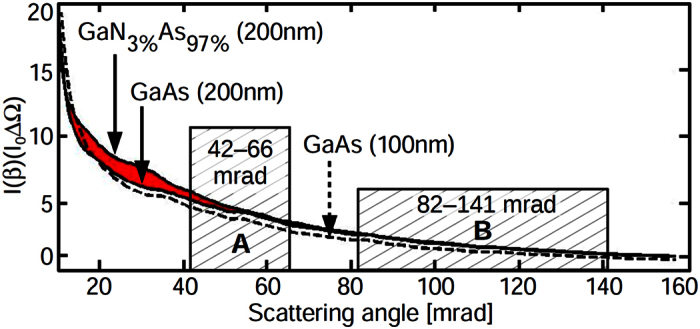
Angular dependence of scattered intensity for GaN_x_As_1−x_. This simulation depicts the scattered intensity for GaAs and GaNAs at different thicknesses, normalised to the incident beam intensity *I*_0_ and the solid angle ΔΩ. The ranges of the ADF detector used for Fig. 2 are marked as A and B. Substitution of only 3% of As by N causes high static disorder which leads to a strong effect (shown red) in the angular dependence owing to Huang scattering.

**Figure 2:**
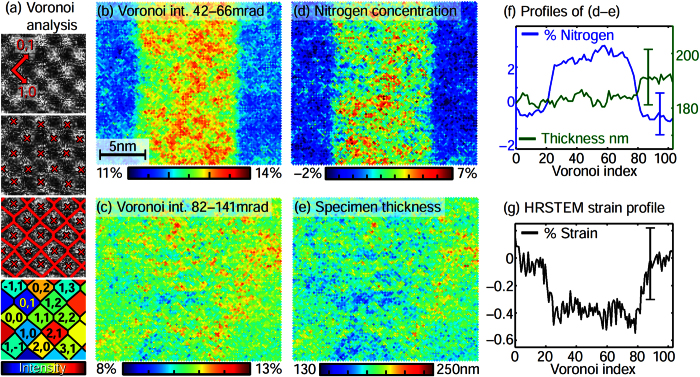
Quantitative evaluation of specimen thickness, composition and strain in GaN_x_As_1−x_ at atomic resolution. (**a**) Construction of Voronoi diagrams from high-resolution STEM images. (**b,c**) Voronoi images for the angular ranges 42–66 and 82–141 mrad. Intensity is given in fractions of *I*_0_. Comparison with simulation yields (**d**) nitrogen content and (**e**) specimen thickness for each atomic column. (**f**) Profiles from averaging the data in (**d,e**) vertically. (**g**) Strain profile measured from the atomic column distances in the STEM image with largest acceptance angle.

The simulated angular dependence of the scattered intensity in Fig. 1 emphasises the GaN_x_As_1−x_/GaAs system as a paradigm for composition- and thickness-specific angle intervals. The scattered intensity I(*β*) has been normalised to the intensity *I*_0_ of the incident electron beam and to the solid angle ΔΩ. Obviously the signal below 60 mrad is sensitive to both nitrogen content and specimen thickness whereas nitrogen hardly affects angles larger than 80 mrad. The signal in the shaded regions A, B has been used to measure thickness and composition independently. To this end, four 2 K × 2 K atomic-resolution images with iris radii of 42, 66, 82 and 141 mrad were recorded at an FEI Titan 80/300 (S)TEM microscope operated at 300 kV using a camera length of 300 mm. As illustrated in Fig. 2a atomic columns were detected in each image, from which a Voronoi diagram was calculated so as to average the intensity within the Voronoi cells, now indexed with respect to the atomic columns. The four Voronoi diagrams were correlated to compensate for specimen drift amounting to eight cells at maximum between the first and the last image, respectively. (See Supplementary Fig. S1 for the detailed data analysis).

By virtue of equation (1) the Voronoi diagrams for the angular ranges A, B in Fig. 1 were calculated yielding the Voronoi intensities for 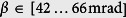 in Fig. 2b and for 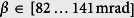 in Fig. 2c. Indeed, Fig. 2b exhibits the GaNAs layer with high contrast to the GaAs buffer. Remarkably, the same region in Fig. 2c does not provide chemical contrast at all. Hence utilising both signals in Fig. 2b,c enables us to determine the local specimen thickness and the local nitrogen content independently by the simultaneous comparison with composition- and thickness-dependent simulations^1^,^2^. In particular, frozen phonon^23^ multislice simulations^24^ account for Huang scattering^25^ caused by the small covalent radius of nitrogen as worked out previously^4^,^26^,^27^,^28^, thermal diffuse scattering within the Einstein model^23^ and the nonuniform detector response^29^.

The resulting composition and thickness maps are presented in Fig. 2d,e with profiles in Fig. 2f. The thickness of 186 nm is nearly uniform whereas the nitrogen content in the layer takes values of 2.5–3%. Given the precision of 0.8% measured in terms of the standard deviation in GaAs and the additional random-alloy fluctuation of 0.4% in GaNAs, this result is in accordance with former STEM and X-ray studies^4^. We stress that both thickness and composition are measured atomically resolved and independently, whereas existing STEM approaches derive the thickness by interpolation from regions with known composition^2^,^16^. Preferential etching during specimen preparation would thus not be noticed and specimen areas with known composition must be present in the image, which is difficult for larger, complex structures. In contrast, recording solely the GaNAs layer would have sufficed in our present approach. We finally augment our analysis by the strain measurement in Fig. 2g which has been performed in the image with the largest iris radius of 141 mrad after Wiener noise filtering and refining atomic column positions with subpixel accuracy. The lattice constant in the GaNAs layer drops by 0.5 ± 0.3 % confirming the nitrogen concentration profile.

### Contrast formation in a MOSFET device

**Figure 3:**
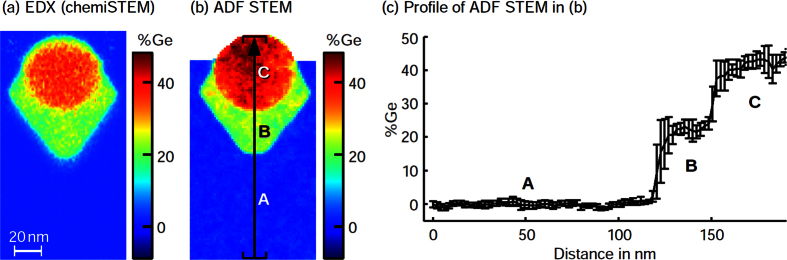
Composition quantification of the Ge_x_Si_1−x_ MOSFET. Ge map of one stressor in a MOSFET obtained from (**a**) EDX and (**b**) HAADF-STEM. The concentration profile (**c**) along growth direction [001] was taken along the black path. Three concentration regimes are present with 0% (A), 22% (B) and 37% (C) Ge.

**Figure 4:**
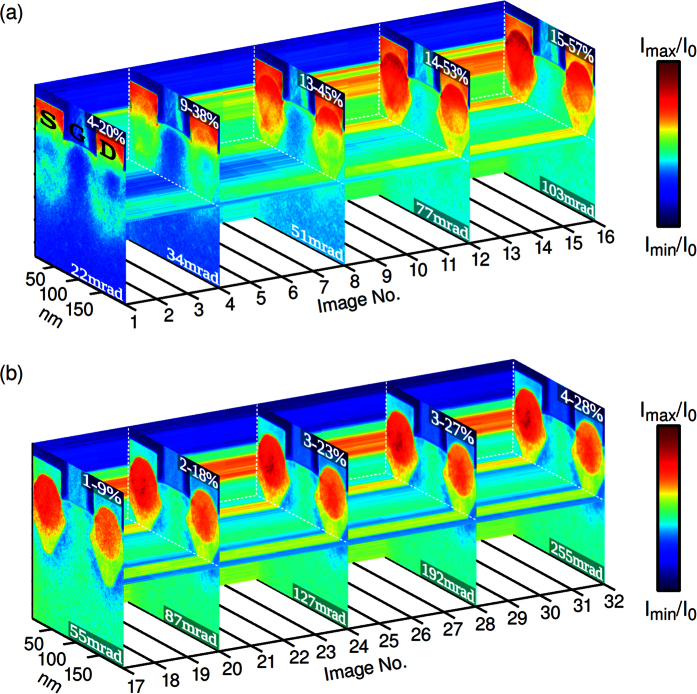
ARSTEM series of the Ge_x_Si_1−x_ MOSFET. The displayed set of STEM images (selection) was taken at camera lengths of (**a**) 478 and (**b**) 195 mm with different radii of the iris aperture as indicated at the bottom of each image. Source S, drain D and gate G are marked in image 1 in (**a**). The inner acceptance angle is (**a**) 16 and (**b**) 35 mrad. The colour-coded signal of each image was normalised to the intensity *I*_0_ of the incoming beam with limits given at the top of the images.

We now turn towards an ARSTEM study of a Ge_x_Si_1−x_/Si MOSFET device in which the Ge content *x* has initially been measured by energy-dispersive Xray (EDX) analysis and quantitative STEM^2^,^30^ using the full HAADF detector range of 35–255 mrad. The composition for one stressor region is shown in Fig. 3, exhibiting two Ge regimes with *x*_B_ = 22 ± 2% and *x*_C_ = 37 ± 3%. Conceptually, a pair of stressors is supposed to compress the Si in between owing to a lattice mismatch of 4% between Ge and Si to enhance carrier mobility in the gate channel. In order to obtain both thickness- and composition-dependent data, two nominally identical MOSFETs from the same fabrication have been thinned by focussed ion-beam preparation to thicknesses around 50 and 150 nm. ARSTEM series were recorded at two different camera lengths of 478 and 195 mm in the angular range [16 … 255 mrad]. A selection of images of the thicker region is depicted Fig. 4. The colour expresses the intensity normalised to that of the incident electron beam, *I*_0_, with limits specified at the top and the iris aperture radius given at the bottom of each image.

The first image in Fig. 4a for *β* ∈ [16 … 22 mrad] completely lacks chemical contrast in favour of strain-dominated intensity modulations in the vicinity of the Ge-containing source S and drain D stressors. Towards image 4 for *β* ∈ [16 … 34 mrad] strain and the onset of *Z*-contrast determine the image contrast comparably. The latter is due to the 2 times larger atomic number of Ge (*Z* = 32) compared to Si (*Z* = 14). In images 8, 12 and 16 for *β* ∈ [16 … 51 mrad], *β* ∈ [16 … 77 mrad] and *β* ∈ [16 … 103 mrad] *Z*-contrast dominates the signal revealing the two regimes of Ge-content within the stressors. This feature continues in subsequent images 17–32 in Fig. 4b recorded at a smaller camera length to cover an angular range of 35–255 mrad. However, strain contrast appears again, but now as a deficiency of intensity around the stressor edges. Since in strained Si scattering into the interval *β* ∈ [16 … 22 mrad] is enhanced with respect to unstrained Si, this will cause a deficiency of intensity in strained regions in STEM images taken with inner acceptance angles larger than these values.

### Verification of scattering theories

**Figure 5:**
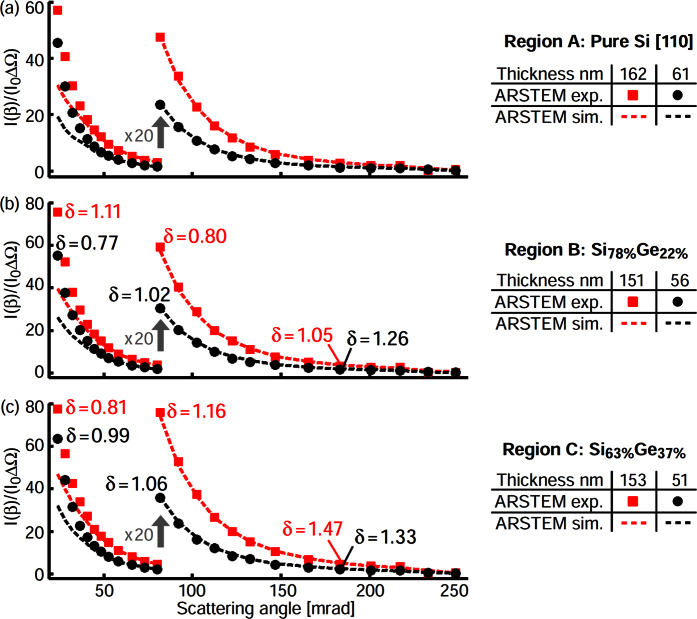
Scattered intensity in dependence of angle, specimen thickness and Ge content. The data was calculated in the three regimes A,B,C marked in Fig. 3b, corresponding to (**a**) pure Si, (**b**) Ge content *x*_B_ = 22% and (**c**) Ge content *x*_C_ = 37%. Red/black data refer to the thick/thin specimen. Dashed lines represent (multislice) simulations. *δ* is the exponent for the modified Rutherford model for selected angles.

Furthermore the dense angular sampling of the MOSFET data allows for the quantitative, angle-dependent analysis presented by the circles and squares in Fig. 5. In particular, eq. (1) was applied to subsequent images of the series in Fig. 4 and to the analogous series for the 50 nm thick specimen. This yields *I*(*β*)/*I*_0_, the intensity scattered on a virtual annular detector with inner and outer acceptance angles determined by two subsequent iris aperture radii in fractions of *I*_0_. Additional normalisation to the solid angle ΔΩ covered by the detector makes the signal independent of the detector area. The simulated counterpart is depicted by dashed lines. Figure 5a corresponds to the average in a 20 × 20 nm^2^ region in pure, unstrained Si labeled A in Fig. 3b. Signal averages from alloy regions labeled B and C in Fig. 3b yield the ARSTEM data Fig. 5b,c.

Comparison of the thick (red) and the thin (black) specimen exhibits that a thickness increase of approximately 100 nm enhances scattering to angles beyond 70 mrad. Here the intensity per solid angle is more than a factor of 2.2 higher for the red than for the black data whereas the ratio drops to 1.2 for angles around 20 mrad. This is expected from scattering theory as high-angle scattering is dominated by thermal diffuse intensity emerging with increasing thickness.

It is instructive to compare the experimental ARSTEM curves in Fig. 5 with theory. During the early development of STEM, the term *Z-contrast* was established for element-specific signals captured at large scattering angles by reference to Rutherford’s scattering theory as the simplest approach. Although this approach neglects charge screening and structural effects, it is still frequently used to interpret experiments, usually in conjunction with the replacement of the quadratic dependence on atomic number *Z* by a modified *Z*^*δ*^ dependence. The broad variety of values for *δ* between 1.5 and 2^31^,^32^,^33^,^34^,^35^ indicates it to be a rather ambiguous parameter. By deriving an effective atomic number^36^ for Ge_x_Si_1−x_ according to 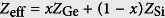, *δ* was calculated in dependence of scattering angle, thickness and composition as exemplarily noted in Fig. 5b,c. The variety of *δ* given shows that there is no consistent trend with respect to a composition, thickness and angle-dependence. We thus conclude that the Rutherford theory is inapplicable to interpret STEM data quantitatively in the present angular range below 255 mrad, though it might be applicable to electron backscattering^37^. In agreement with this finding, the composition quantification in Ge_x_Si_1−x_ via Rutherford’s approach could rather be accomplished by extrapolating angle-dependent data to scattering angles of π/2 than by explicit evaluation of images taken at different camera lengths^36^.

An advanced modelling of HAADF STEM intensities is possible by multislice simulations based on the solution of the relativistically corrected Schrödinger equation including thermal and static disorder. Our ARSTEM experiment permits an explicit angle-dependent comparison with simulations shown as dashed lines in Fig. 5. The simulation is in perfect agreement with experiment for both thicknesses and all three compositions - except for angles smaller than approximately 35 mrad. Here the simulation underestimates the scattered intensity by a factor of up to 2.3. However, the excellent large-angle agreement indicates that the complex concurrence of phonon-, Huang-, multiple scattering, propagation of the electron wave in the specimen and the scattering factors used^38^ is treated correctly. Note that matching simulation and experiment in the low-angle regime would lead to specimen thicknesses and compositions disagreeing with former studies^1^,^2^,^39^,^30^ and the composition analysis in Fig. 3a,b. A discussion of the inconsistency between contemporary multislice simulations and low-angle scattering will be the major subject of the next section.

## Discussion

The introduction of an aperture with variable radius into the field of TEM offers a degree of freedom for versatile STEM imaging as to the visualisation of strain fields, thickness contrast, Huang scattering and Z-contrast. Though our sequential approach possibly requires correlation of subsequent images to compensate for specimen drift, it allows for acquisition times dedicated for the angular range of interest and the scanning of single 2 K × 2 K STEM images (or larger) with common frame times, which cannot be achieved with, e.g., contemporary ultrafast cameras^13^,^15^,^40^. Aside from that, the cross-correlations involved for a whole ARSTEM series are basically a standard procedure routinely used to analyse (S)TEM data immediately after acquisition within a few minutes. Moreover, we demonstrated that quantitative, atomic resolution mapping of composition, thickness and strain is possible by adjusting detector acceptance angles according to the angular ranges most sensitive to the quantity of interest derived from simulations in this case. This is a promising approach for composition mapping in more complex structures, e.g., alloyed nanoparticles where the thickness along electron beam direction often varies drastically.

We finally discuss the mismatch at low angles by examining to which extent established simulations as the present ones reflect the experimental conditions. Surface contamination with hydrocarbons or amorphous Si/Ge (oxide) layers could make an effect, but then one expects a decrease of the mismatch with increasing specimen thickness. More importantly, one must keep in mind that contemporary simulations include elastic scattering only, aside from phonon excitations. Deviations between simulation and experiment are hence expected if (i) the angular distribution of further inelastic scattering differs from the elastic one and (ii) if the inelastic intensity amounts to a significant fraction of *I*_0_.

Concerning (i) the (differential) inelastic cross section in dipole approximation obeys a Lorentzian proportional to 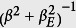 with the characteristic scattering angle *β*_*E*_ = Δ*E*(*γmv*^2^)^−1^ being equal to the half width at half maximum. The fact that *β*_*E*_(100 eV) ≈ 0.2 mrad and *β*_*E*_(20 eV) ≈ 0.04 mrad yield very narrow Lorentzians for typical Si core and plasmon excitations for an acceleration voltage of 300 kV, respectively, has frequently been a leading argument for assigning diffuse intensity in diffraction patterns solely to phonon scattering. On top of that, the inelastic cross section decreases even stronger at higher angles than predicted by the Lorentzian form. However, several studies as to the angular distribution of inelastic scattering pointed out both theoretically^41^,^42^,^43^ and experimentally^44^,^45^ that diffuse intensity originates significantly from inelastic scattering, predominantly from electronic excitations that are collective in nature. For inner shell excitations the Lorentzian shape is approximately valid up to the Bethe ridge angle of approximately 20 mrad here. In the case of plasmon scattering the Lorentzian form is correct up to approximately 4.5 mrad, the critical angle for plasmon scattering^46^ which is the dominant inelastic process. Hence inelastic scattering is found at the order of Bragg angles and beyond, with an explicit angular distribution for each energy loss. Deviations from the angular distribution of elastic and phonon scattering likely arise from these tails of the Lorentzian that lead to a significant fraction of diffuse intensity in the diffraction pattern which has indeed been found comparable to phonon contributions for Si^45^. This is because the tails of a narrow Lorentzian for the differential cross section can amount to significant values after integration over the solid angle of interest.

As to (ii), our experiments are performed at thicknesses in the order of the mean free path for inelastic scattering, which hence makes a significant fraction of *I*_0_. As an experimental proof we recorded zero-loss energy filtered and unfiltered diffraction patterns in pure Si. Indeed, strong dependence of the ratio of elastic to inelastic intensity of the scattering angle was observed up to 35 mrad (Supplementary Note and Supplementary Fig. S4). We note that these findings agree with previous reports in which low-angle unfiltered STEM served for qualitative analyses in thick specimen^47^,^48^,^49^ whereas quantitative agreement was found in bright field STEM in thin specimens where the fraction of inelastic intensity is low^50^. Moreover, energy filtered diffraction patterns at elevated thicknesses agreed with simulations, too^51^,^52^,^53^.

In conclusion, we exploited the angular resolution in STEM as a free experimental parameter. The benefits of angle-resolved STEM have been demonstrated by means of the independent quantification of composition, specimen thickness and strain in GaN_x_As_1−x_, by the ability to visually distinguish contrast due to composition and strain in a Ge_x_Si_1−x_/Si MOSFET, and by enabling the quantitative test of established theoretical models for the description of the angle-dependent scattered intensity. Namely the Rutherford model is inconsistent with our experiments ranging up to 255 mrad while multislice frozen phonon simulations are, except for angles below 35 mrad, for which the influence of further inelastic scattering was made responsible. Our setup overcomes drastic limitations owing to the speed of slow-scan pixelated detectors that have as yet allowed for spatially resolved diffractometry on a STEM raster of 64 × 64 pixels^54^, which restricts the field of view to the scale of approximately one crystal unit cell in high-resolution STEM. Considering former semi-quantitative STEM studies that were aiming at the determination of two different chemical compositions^47^ and at eliminating contrast arising from surface strain fields^49^ using two different camera lengths, the present option of imaging at dedicated angle intervals is expected to improve the quantitative composition analysis in established semiconductor nanostructures significantly. Furthermore, recent studies^55^ employing variable-angle STEM by means of two camera lengths and a conventional annular detector have successfully demonstrated the detection mapping of dopant atoms in 3D as a proof of concept. However, detector acceptance intervals covering at least 250 mrad had been used to capture changes in the angular dependence of scattered intensity at a scale of 30 mrad and below^55^ which demonstrates the potential of the approach presented here concerning further contemporary challenges in materials science.

## Methods

### Experimental details

The FEI Titan 80/300 (S)TEM microscope operated at 300 kV has a spherical aberration constant of 1.2 mm of the probe forming lens, so that Scherzer conditions used here correspond to a convergence semi-angle of 9 mrad and a defocus of −48 nm. A spot number setting of 9 was used with an extraction voltage of 4500 V and a gun lens setting of 6. The Fischione Model 3000 annular detector amplifiers have been set such that the detector operates in the linear range. All specimens have been prepared by focused ion beam (FIB) milling using Ga ions, followed by a low-energy milling step using Ar ions with an energy of 800 eV to remove amorphous surface layers induced by the FIB preparation. Eventually plasma cleaning using an ArO plasma was performed immediately before the STEM experiments.

The iris aperture (Supplementary Fig. S3) is placed directly above the HAADF detector. We used a common TEM aperture bellow with lateral xy-translation to mount the iris frame at the 36 mm port of the Titan 80/300 microscope, opposite to the HAADF detector port. The 36 mm flange is situated above the viewing chamber directly below the differential pumping aperture. The opening range of the iris covers the full size of the HAADF detector. The aperture itself was developed by the company Sahm Feinwerktechnik GmbH (Friedensstr. 3, 35580 Wetzlar, Germany) and consists of 16 lamellae in double-layer arrangement enabling both a full opening of max. 36.7 mm and a full shutting. The aperture is connected to an ultrahigh vacuum stepper motor Model VSS 19.200.0,6-HV-NSSN-4Lp fabricated by Phytron GmbH (Industriestr. 12, 82194 Gröbenzell, Germany) with a maximum torque of 3.4 mNm. Components of this motor have been magnetised such that magnetic stray fields are eliminated. The gear wheel was chosen such that the aperture closes/opens within 260 integer steps of motion of the stepper motor, which can optionally be subdivided into 256 substeps. Here only integer step intervals were used. For example, one integer step corresponds to approximately 1.1 mrad at a camera length of 195 mm.

The intensity *I*_0_ of the incoming STEM beam was measured from conventional “detector scans”^2^,^29^,^56^ (Supplementary Fig. S1: detector scans for the GaNAs study), which are acquired by scanning the primary beam over the high-angle annular dark field detector (Fischione 3000) in imaging mode without specimen. For a quantitative comparison, simulations must account for the inhomogeneous sensitivity of the detector which can be done in two ways. First, azimuthal averages are calculated from the detector scans just mentioned, being the conventional method to obtain the radial sensitivity^2^. Second, the incident STEM beam is tilted systematically while the microscope is set to diffraction mode so as to cover the angular domain transferred by the imaging system^56^. Such tilt-based detector scans fully include all distortions of the diffraction pattern caused by the aberration corrector for imaging. Because the tilt-based method can be time consuming for the multitude of iris radii used in the presented studies, we checked whether it leads to different results compared to using conventional detector scans for some representative angles. For each iris radius of the GaNAs study, 256 × 256 beam tilts up to a tilt angle of 320 mrad have been used on a regular square raster. Radial detector sensitivities are found to be practically identical, except that the tilt-based method reveals weak contributions of electrons scattered to an interval 210–250 mrad which are bent back onto the detector for the intended angular range 42–141 mrad (Supplementary Fig. S2). However, for 210–250 mrad the average sensitivity is only 0.06, and Fig. 1 indicates insignificant scattering there in comparison to the 42–141 mrad interval. Consequently, the treatment of the inhomogeneous detector sensitivity in the conventional way is found to modify the nitrogen content (Fig. 2d) by 0.1% at maximum and the specimen thickness (Fig. 2e) by less than 4 nm which is well below the experimental error margins. As to the MOSFET study in Figs 4 and 5, distortions of the diffraction pattern have been found to influence scattering beyond 200 mrad (camera length 195 mm), where again the signal is extremely low. Particularly the discussion of discrepancies between experiment and theory at much lower angles is not affected by this issue.

The reproducibility of setting a desired aperture radius was verified by taking two subsequent detector scan series, one starting at the fully closed aperture while subsequently increasing the radius in integer steps, the other vice versa. No significant difference was observed in azimuthal averages of the intensities. This was achieved by tensely mounting the gear of the stepper motor with respect to the geared rim of the iris. Further details on the implementation of ARSTEM data acquisition and analysis as well as the evaluation of the GaNAs and MOSFET data and the acquisition of the tilt-based detector sensitivities are briefly described in Supplementary Methods.

### Simulation details

We used the STEMsim^24^ software to simulate thickness-, composition- and angle-dependent STEM intensities for the cubic materials GaN_x_As_1−x_/GaAs and Ge_x_Si_1−x_/Si. Simulation details^30^,^49^ are summarised in brief here. The incident 300 kV STEM probe was simulated using a spherical aberration of *C*_*s*_ = 1.2 mm for the probe-forming SuperTwin objective lens of the microscope at Scherzer defocus of −48 nm and Scherzer aperture corresponding to a convergence semi-angle of 9 mrad. The probe was then propagated through the specimen using the multislice frozen phonon approach assuming uncorrelated vibration of the atoms according to the Einstein model. The LAMMPS^57^ software was used to calculate static disorder due to different covalent radii of substitutional nitrogen or germanium atoms in 5 × 5 (GaNAs) and 7 × 7 (GeSi) supercells with 200 nm thickness in electron beam direction which was [001] for GaNAs and [110] for GeSi. The LAMMPS code minimizes strain energy by using empirical potentials proposed by Keating^26^,^28^ (GaNAs) and Tersoff 58 (GeSi) to calculate static atomic displacements which cause additional diffuse intensity by virtue of Huang scattering. The supercells have been strained to account for pseudomorphic growth of alloy layers on the GaAs and Si substrate with tetragonal distortions derived from elasticity theory. For each probe position we calculated the azimuthal sum of scattered intensity which was then averaged over 20 × 20 (GeSi) or 15 × 15 (GaNAs) probe positions in one unit cell. Furthermore, thermal and compositional configuration averaging was performed over 20 supercells in which the distribution of substitutional atoms and thermal displacements have been varied according to random alloys and Gaussian statistics, respectively. For the latter we used Debye parameters *B* = 8π^2^〈*u*^2^〉 with 〈*u*^2^〉 the mean squared displacement of the atoms calculated from density functional theory^59^ according to 300 K. For Ga bound to As this gives *B*_Ga_ = 0.73 Å^2^ and *B*_As_ = 0.62 Å^2^, for Ga bound to N we have *B*_Ga_ = 0.25 Å^2^ and *B*_N_ = 0.29 Å^2^. Furthermore we used *B*_Si_ = 0.53 Å^2^ and *B*_Ge_ = 0.67 Å^2^. See Supplementary Figures S5 and S6 for the simulated Voronoi intensities of GaNAs and GeSi, respectively.

## Additional Information

**How to cite this article**: Müller-Caspary, K. *et al*. Materials characterisation by angle-resolved scanning transmission electron microscopy. *Sci. Rep.* **6**, 37146; doi: 10.1038/srep37146 (2016).

**Publisher’s note**: Springer Nature remains neutral with regard to jurisdictional claims in published maps and institutional affiliations.

## Supplementary Material

Supplementary Information
