# Supplementary material for: Materials characterisation by angle-resolved scanning transmission electron microscopy
Source: Sci Rep. 2016 Nov 16;6:37146. doi: 10.1038/srep37146 (PMC5111052; doi:10.1038/srep37146)
Supplement: Supplementary Information [file srep37146-s1.pdf]

Supplementary Information for:

Materials characterisation by angle-resolved scanning transmission electron microscopy

Knut Müller-Caspary[1], Oliver Oppermann[1], Tim Grieb[1], Florian F. Krause[1], Andreas Rosenauer[1], Marco Schowalter[1], Thorsten Mehrtens[1], Andreas Beyer[2], Kerstin Volz[2], and Pavel Potapov[3]

[1]Institut für Festkörperphysik, Universität Bremen, Otto-Hahn-Allee 1, 28359 Bremen, Germany
[2]Faculty of Physics and Materials Science Center, Philipps Universität Marburg, Hans-Meerwein-Straße, 35032 Marburg, Germany
[3]GLOBALFOUNDRIES Dresden Module 1, Wilschdorfer Landstraße 101, 01109 Dresden, Germany

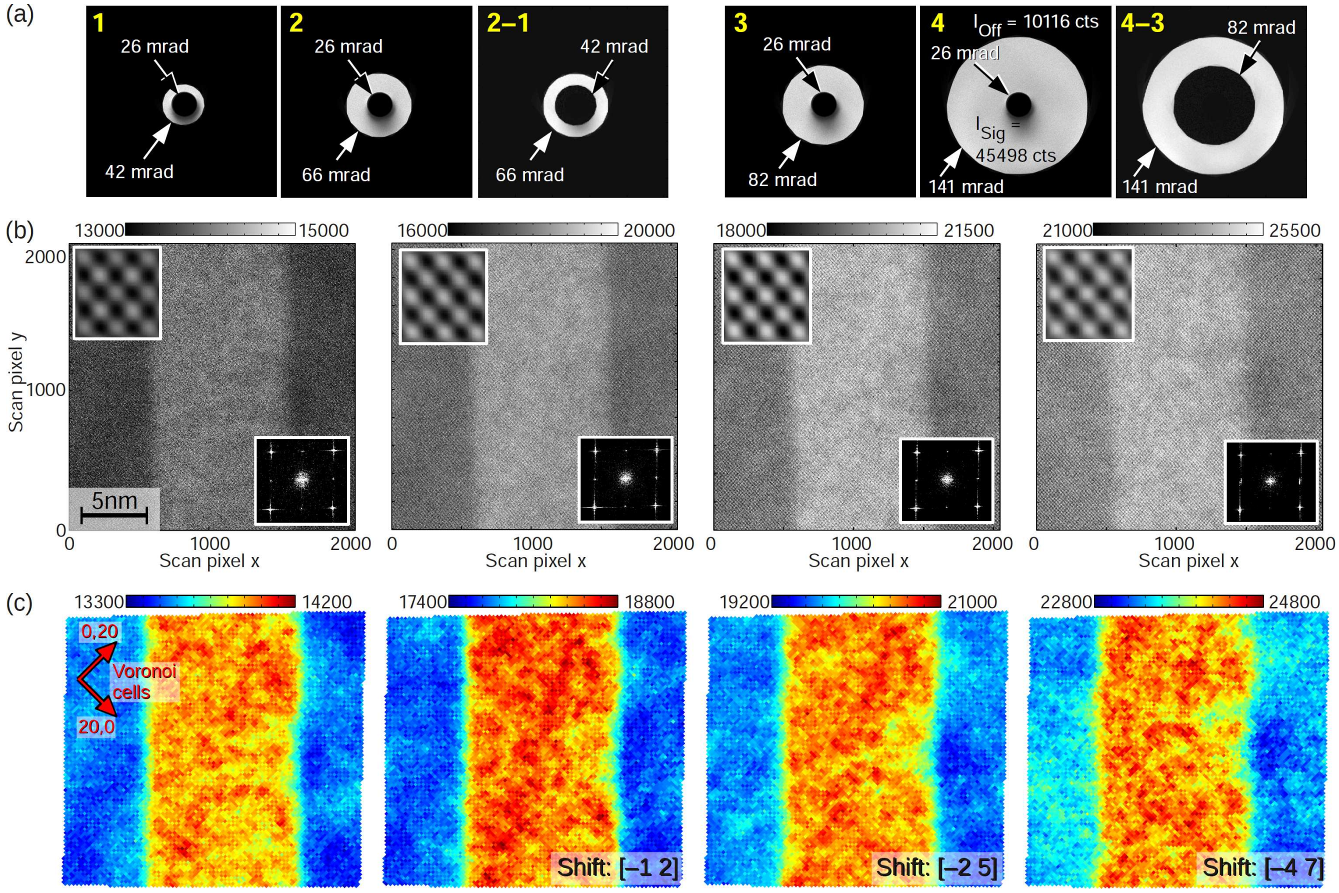

Supplementary Figure S 1. Data processing for the analysis of GaNAs. (a) Raw detector scans (1,2,3,4) illustrating the iris radii used to record STEM images for the angular ranges A and B in Fig. 1. The differences 2 – 1 and 4 – 3 show the effective ADF detectors covering the ranges A, B. No. 4 contains the signal and offset levels used to determine I_0. (b) Raw 2K×2K atomic resolution STEM images of the GaNAs layer for angle intervals 26 – 42, 26 – 66, 26 – 82 and 26 – 141 mrad. The power spectra in the insets at the bottom indicate the atomic resolution. Insets at the top show parts of the filtered images used to detect atomic columns for the subsequent Voronoi segmentation of the unfiltered images, and for strain state analysis (rightmost image). (c) Voronoi diagrams calculated from the images in (b), including the shifts relative to the leftmost Voronoi diagram. The difference between the two diagrams on the left yields Fig. 2 b, the difference between the two diagrams on the right yields Fig. 2 c after normalisation to I_0.

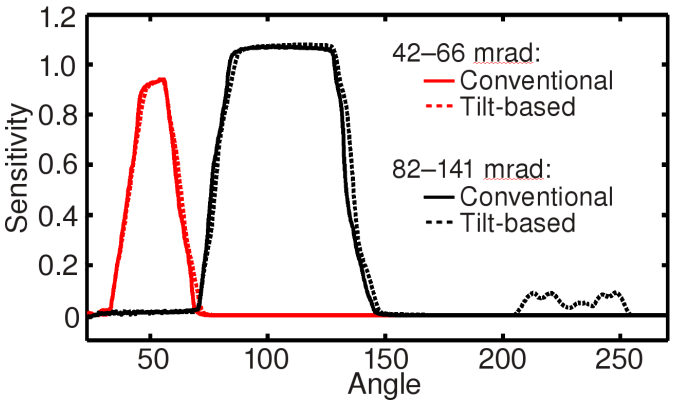

Supplementary Figure S 2. On the influence of distortions of the diffraction pattern on detector sensitivities. Comparison of azimuthal averages of the detector sensitivities obtained by scanning the STEM beam over the detector in imaging mode (conventional method, solid) and by tilting the incident STEM beam in diffraction mode (dashed). The former correspond to the data in Supplementary Fig. S 1a. Within the two angular intervals 42 – 66 mrad and 82 – 141 mrad the sensitivities are practically identical, whereas the tilt-based method reveals a small effect of large angle scattering (210 – 250 mrad) bent back onto the detector, which was checked to leave the results in Fig. 2 unchanged within the experimental precision.

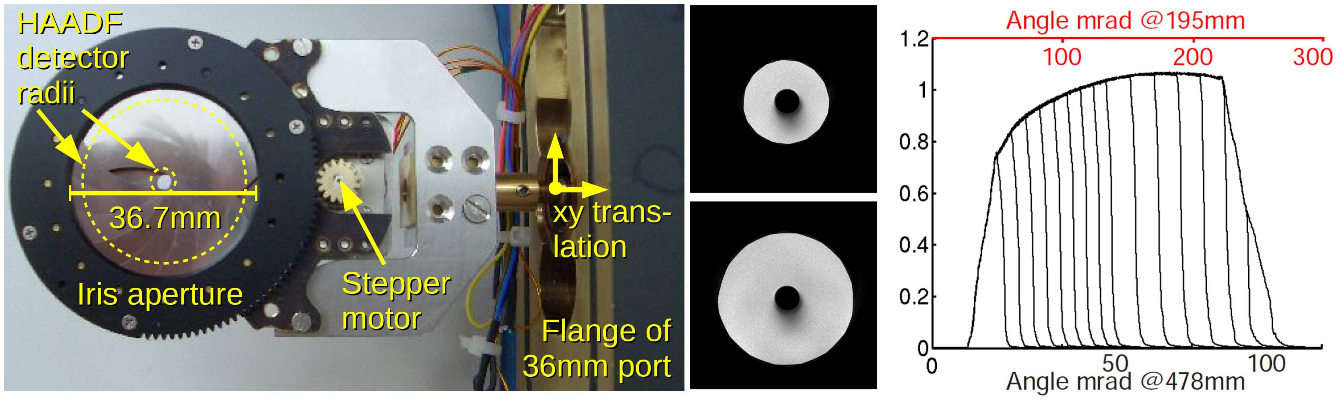

Supplementary Figure S 3. The iris aperture. *Left:* Photograph of the iris aperture (top view) with the gear of the stepper motor and xy translation employing a conventional TEM aperture drive for centering the aperture over the HAADF detector (dashed circles: inner/outer rim). The setup is mounted to a flange for the 36 mm port of the Titan microscope, opposite to the HAADF detector. *Centre:* Two example detector scans at different iris radii. *Right:* Radial sensitivity curves for the ARSTEM series in Fig. 4 (for both camera lengths) to account for the nonuniform detector response in simulations.

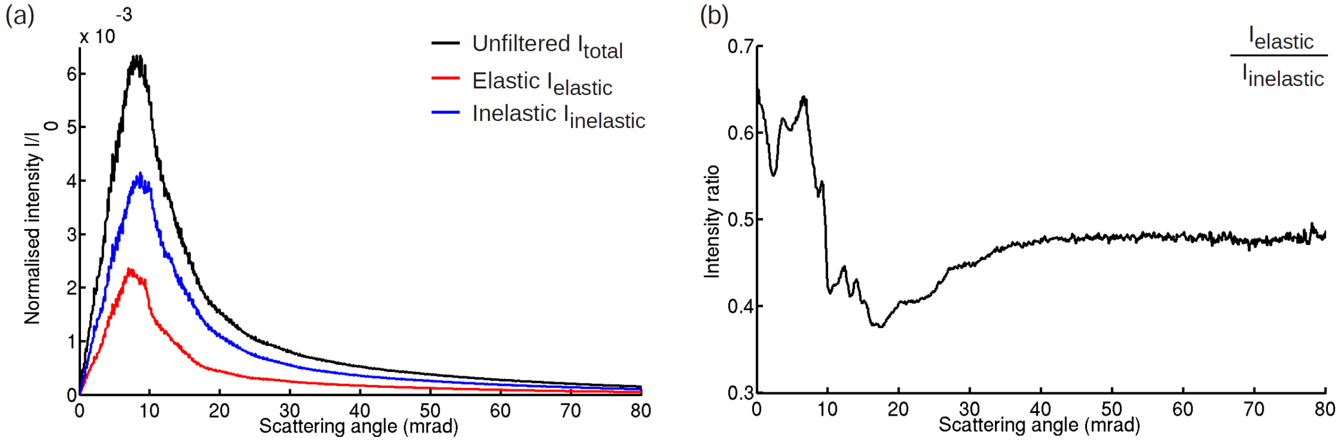

Supplementary Figure S 4. The influence of inelastic scattering. (a) Angular dependence calculated from position-averaged convergent beam electron diffraction (PACBED) patterns recorded on CCD using a Gatan Imaging Filter (GIF). Azimuthal averages of the elastic (slit width 10 eV), the total (i.e. unfiltered) and the inelastic intensity are shown. All three curves are normalised to the sum of I_{total}. (b) Ratio of the red and blue data in (a) as a function of the scattering angle. From this it can be concluded that it is necessary to take the individual angular dependence of inelastic scattering into account in simulations for low angles < 35 mrad.

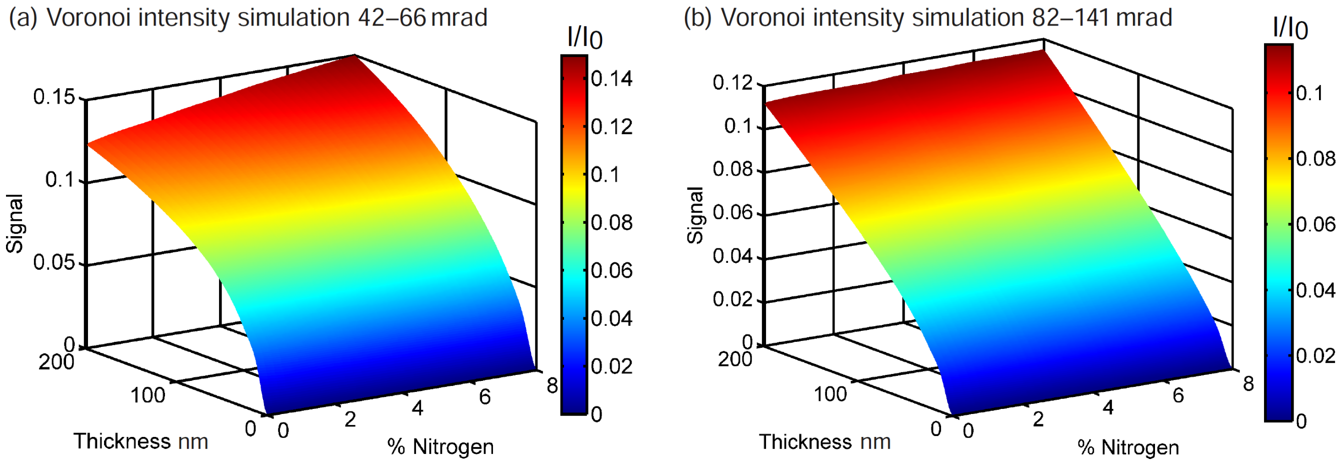

Supplementary Figure S 5. Simulated Voronoi intensities for GaNAs. Result for (a) detection angles 42 – 66 mrad and (b) 82 – 141 mrad as a function of the specimen thickness and the nitrogen content. These simulations correspond to the angular intervals used in experiment marked as A and B in Fig. 1, respectively. The nonuniform detector sensitivity is included. Figures 2 b,c have been compared directly with this simulation.

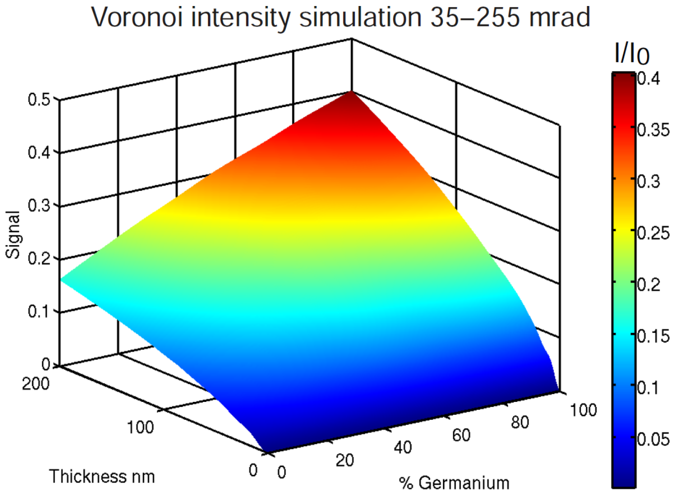

Supplementary Figure S 6. Simulated Voronoi intensities for GeSi. The simulation corresponds to the conventional STEM setting at a camera length of 195 mm and retracted iris aperture, i.e. detection angles of 35 – 255 mrad. The nonuniform detector sensitivity is included. To determine the germanium map in Fig. 3 b from ADF STEM, this data was used. In particular, a polynomial surface of order 2 has been fitted to regions of pure Si outside the Ge stressor, from which the specimen thickness throughout the whole region of interest was determined.

Supplementary Note: Angular dependence of inelastic scattering

In the manuscript we discussed the different angular dependencies of inelastically and elastically scattered electrons as a major reason for the mismatch of simulation and experiment at angles below 35 mrad, for what we present the experimental evidence here. A Tridiem Gatan Imaging Filter (GIF) Model 863 was used to record three zero-loss energy filtered diffraction patterns of Si in [110] zone axis orientation with a slit width of 10 eV. In addition, we recorded three unfiltered diffraction patterns with the energy-filtering slit retracted. While recording the images, the STEM beam was scanned over an approximately 10×10 nm large region which gives a position-averaged convergent-beam electron diffraction pattern (PACBED). Images have been recorded on a Gatan UltraScan 1000 camera with a size of 2K×2K pixels with gain correction and dark subtraction. The zero-loss images have been integrated over 41 s, the unfiltered ones over 20.5 s. Each three zero-loss/unfiltered images have been summed up to gain better statistics at higher angles. The unfiltered PACBED patterns have been corrected for the different illumination time by multiplication with 2. Finally, the intensity was summed azimuthally which yields the dependence of the elastic (I_{elastic}) and the total (I_{total}) intensity on the scattering angle. Moreover, the difference between both was also calculated which equals the angular dependence of the inelastic intensity $I_{\text{inelastic}}$. The specimen thickness in the PACBED region was determined to 79 ± 3 nm by conventional HAADF-STEM, i.e. using detector acceptance angles of $35-255$ mrad and comparing with the simulation for pure Si as shown in Supplementary Fig. S 6.

The three curves I_{elastic}, I_{total} and $I_{\text{inelastic}}$ have been normalised to the sum of I_{total} (which is $> 0.99\, I_0$) and are depicted in Supplementary Fig. S 4 a. Obviously the inelastic intensity approximately amounts to twice the elastic intensity (except for phonon scattering which is still present in I_{elastic} due to its extremely low energy loss). The ratio $I_{\text{elastic}}/I_{\text{inelastic}}$ is depicted in Supplementary Fig. S 4 b in dependence of the scattering angle. Clearly this ratio depends strongly on angle below $35-40$ mrad. For example, the ratio is approximately 2 times higher within the primary beam, i.e. in the range $0-9$ mrad, than in the angular range of $15-25$ mrad.

Interestingly, the ratio remains constant beyond $35-40$ mrad. This means that inelastic and elastic scattering must be treated separately below these angles, but not above them, at least concerning the azimuthal integrals. In addition, it is expected that each energy loss has its individual angular dependence which makes the quantitative simulation at low angles a challenging (future) task.

Supplementary Methods

ARSTEM implementation. Both acquisition and evaluation of ARSTEM data has been implemented in our Matlab-based *ImageEval* software. In particular, setting the iris aperture to a desired radius via RS232-based communication with the stepper motor controller and acquiring a STEM image via common object model (COM) based communication with the microscope system has been automatised. In addition, *ImageEval* enables the conventional quantitative STEM evaluation[1,2], namely normalisation of scattered intensities to the intensity of the incoming beam[3,1], the Voronoi- or cartesian image segmentation and subsequent calculation of average intensities within each segment. For ARSTEM, tools such as the alignment of images to compensate for specimen drift, calculation of arbitrary image differences within an ARSTEM series and multidimensional comparison of experimental data with simulated counterparts has been implemented, e.g., to measure both thickness and composition independently. As to the simulations *ImageEval* has a plugin to read angle-dependent data and to calculate the signal for any annular detector geometry taking into account the nonuniform detector response. The analysis typically takes a few minutes, so that the result is available immediately during the microscope session.

Acquisition and evaluation of the GaNAs data. Supplementary Figure S 1a shows detector scans for the iris radii used. Detector amplifyers were operated in a linear range corresponding to an offset level of $I_{\text{Off}} = 10116$ and a signal level of $I_{\text{Sig}} = 45498$ counts as indicated. Experimental images can then be normalised with respect to the incident beam intensity by subtracting I_{Off} and dividing by $I_0 = I_{\text{Sig}} - I_{\text{Off}}$, which makes them comparable with simulations quantitatively.

The four raw STEM images of the GaNAs layer taken at a camera length of 300 mm are depicted in Supplementary Figure S 1b, corresponding to 2048×2048 scan positions each. The grayscale represents the raw detector signal. Due to the decrease of intensity with increasing scattering angle we used different acquisition times of 50, 70, 90 and 130 s, summing to 5.67 min in total. The insets bottom right show the power spectra verifying the atomic resolution. Atomic columns have been detected in images to which a Wiener noise filter has been applied as illustrated by the insets top left. Note that the Voronoi segmentation has been performed in the unfiltered images, however, with atomic column *positions* from the filtered ones.

The four Voronoi diagrams are shown in Supplementary Figure S 1c, representing the average intensity in each Voronoi cell mapped against the Voronoi index. The three images on the right have been cross-correlated with the first one to determine the relative image shifts which are given at the bottom of each Voronoi diagram. Note that this operation was performed at the coordinate frame of atomic columns and is hence independent of scan noise and drift occurring during the acquisition of a single image. Moreover, each image shows the GaNAs layer with high contrast because Eq. (1), the subtraction of subsequent images, has not been applied yet. Finally, performing the shift correction and subtracting the first from the second and the third

from the fourth Voronoi diagram yields the data in Fig. 2 b,c after normalisation to I_0 as described above. The strain profile in Fig. 2 g has been obtained by using the Wiener noise-filtered image corresponding to an angular range of $26-141$ mrad. Subpixel accuracy was obtained by parabolian fits to the intensity maxima.

Acquisition and evaluation of the MOSFET data. The ARSTEM MOSFET images in Fig. 4 taken at camera lengths of 478 and 195 mm have been acquired and processed in analogy to the GaNAs data, however, with different iris radii given in Fig. 4. Moreover, the field of view with a diameter of approximately 200 nm necessary to capture the characteristic source-gate-stressor region did not allow for atomic resolution imaging as the STEM scan is limited to 2K×2K scan positions. The conventional Z-contrast evaluation in Fig. 3 b was therefore obtained by averaging the experimental image on a cartesian grid with a cell size of 4×4 pixels. Note that this averaging is necessary because of the undersampling of the crystal lattice with an atomically sharp STEM beam in order to compare with Voronoi cell averages in simulations quantitatively.

Acquisition of the tilt-based detector sensitivities. The tilt-based detector scans which result in the sensitivity curves shown in Fig. S 2 were acquired as introduced and described in detail in Ref. 4: A small condenser aperture was used to reduce the diameter of the diffraction disc. Then the beam incident in the specimen plane was tilted using the condenser system deflection coils, while the sample was removed from the beam path. The detector was inserted in diffraction mode and the camera length of interest was chosen. Then for each beam tilt of a regular grid of angles the detector output was recorded. By normalising the resulting image to the incident beam, one gets the tilt-based detector sensitivity that includes all distortions or cut-offs that occur in the imaging and projection system, which forms the diffraction pattern. The acquisition has been automated for FEI microscopes. Due to current software-limitations a highly-resolved tilt-based detector scan as presented here takes four hours, which is why we reduced ourselves to some representative iris radii in the study presented.

Supplementary References

1. Rosenauer, A. *et al.* Measurement of specimen thickness and composition in $Al_xGa_{1-x}N/GaN$ using high-angle annular dark field images. *Ultramicroscopy* **109**, 1171–1182 (2009).

2. Rosenauer, A. *et al.* Composition mapping in InGaN by scanning transmission electron microscopy. *Ultramicroscopy* **111**, 1316–1327 (2011).

3. LeBeau, J. M., Findlay, S. D., Allen, L. J. & Stemmer, S. Quantitative Atomic Resolution Scanning Transmission Electron Microscopy. *Phys. Rev. Lett.* **100**, 206101 (2008).

4. Krause, F. F. *et al.* Effects of instrument imperfections on quantitative scanning transmission electron microscopy. *Ultramicroscopy* **161**, 146–160 (2016).
